# Application of multicolor banding combined with heterochromatic and locus-specific probes identify evolutionary conserved breakpoints in *Hylobates pileatus*

**DOI:** 10.1186/s13039-016-0228-x

**Published:** 2016-02-17

**Authors:** Wiwat Sangpakdee, Alongklod Tanomtong, Xiaobo Fan, Krit Pinthong, Anja Weise, Thomas Liehr

**Affiliations:** Jena University Hospital, Friedrich Schiller University, Institute of Human Genetics, Kollegiengasse 10, D-07743 Jena, Germany; Department of Biology Faculty of Science, Khon Kaen University, 123 Moo 16 Mittapap Rd., Muang District, Khon Kaen, 40002 Thailand; Faculty of Science and Technology, Surindra Rajabhat University, 186 Moo 1, Maung District, Surin, 32000 Thailand

**Keywords:** Multicolor banding (MCB), *Hylobates pileatus* (HPI), *Hylobates lar* (HLA), Evolution, Centromeric positions, Heterochromatin

## Abstract

**Background:**

The question what makes *Homo sapiens sapiens* (HSA) special among other species is one of the basic questions of mankind. A small contribution to answer this question is to study the chromosomal constitution of HSA compared to other, closely related species. In order to check the types and extent of evolutionary conserved breakpoints we studied here for the first time the chromosomes of *Hylobates pileatus* (HPI) compared to HSA and *Hylobates lar* (HLA) by means of molecular cytogenetics.

**Results:**

Overall, 68 new evolutionary conserved breakpoints compared to HSA could be characterized in this study. Interestingly, only seven of those were different compared to HLA. However, application of heterochromatic human DNA-probes provided evidence that observed high chromosomal rearrangement rates of gibbons in HPI happened rather in these repetitive elements than in euchromatin, even though most centromeric positions were preserved in HPI compared to HSA.

**Conclusion:**

Understanding genomes of other species and comparing them to HSA needs full karyotypic and high resolution genomic data to approach both: euchromatic and heterochromatic regions of the studied chromosome-content. This study provides full karyotypic data and previously not available data on heterochromatin-syntenies of HPI and HSA.

**Electronic supplementary material:**

The online version of this article (doi:10.1186/s13039-016-0228-x) contains supplementary material, which is available to authorized users.

## Background

Understanding evolution of human (*Homo sapiens sapiens*, HSA) is one of the major interests of man, latest since Charles Darwin published ‘The origin of species by means of natural selection’ [1]. As Hominoidea superfamily includes great apes (chimpanzees, bonobos, orangutans, gorillas, humans) as well as lesser apes (gibbons; Hylobatidae), studies on chromosomal evolution should include all groups of the superfamily HSA belongs to. For lesser apes it has been shown previously that they underwent high numbers of chromosomal rearrangements including inversions, translocations, fissions and fusions [2] after they divided from their common ancestor with great apes around 15–20 million years ago [3, 4]. The chromosomal rearrangement rate of gibbons was suggested to be at least an order of magnitude higher than the average rearrangement rate in mammals [5, 6], making them an interesting model of evolution.

The family Hylobatidae includes at least 12 species divided into four genera with different constitutional chromosome numbers: *Hoolock* (2n = 38), *Hylobates* (2n = 44), *Symphalangus* (2n = 50) and *Nomascus* (2n = 52) [7]. Besides determining chromosomal numbers and doing basic cytogenetic studies, only a few lesser apes were studied in detail by molecular studies including fluorescence in situ hybridization (FISH). In *Hylobates* there were studied yet by FISH with a whole genomic view *Hylobates lar* (HLA) [8, 9], *H. klossii* [10], *H. moloch* [11], and *H. pileatus* (HPI) [11]. For *H. muelleri*, and *H. agilis* (HAG) it was only shown by now that alpha-satellite sequences as present in HSA are not detectable in those species, including in HLA and HPI [9]. HAG was also studied using a few selected chromosome paints [12]. For *H. albibarbis* genetic studies were not done yet, which still could be helpful to solve the question if it is a subspecies of *H. agilis* [13]. Finally, there are FISH-studies on *H. leucogenys* (now called *Nomascus leucogenys*) [14], *H. concolor* (now called *Nomascus concolor*) [15], *H. syndactylus* (renamed to *Symphalangus syndactylus*) [16].

The pileated gibbon (HPI) has 44 chromosomes and was first karyotyped in 2007 [17]. Yet, detailed molecular cytogenetic characterization was not done, even though high throughput sequencing of its genome was performed recently [11]. Here we report the characterization of 68 evolutionary conserved breakpoints (ECBs) in HPI based on FISH applying high resolution multicolor banding (MCB), locus-specific probes and also all human repetitive probes apart from centromeric ones. The obtained data is an important addition to the already available molecular data.

## Results

**Fig. 1:**
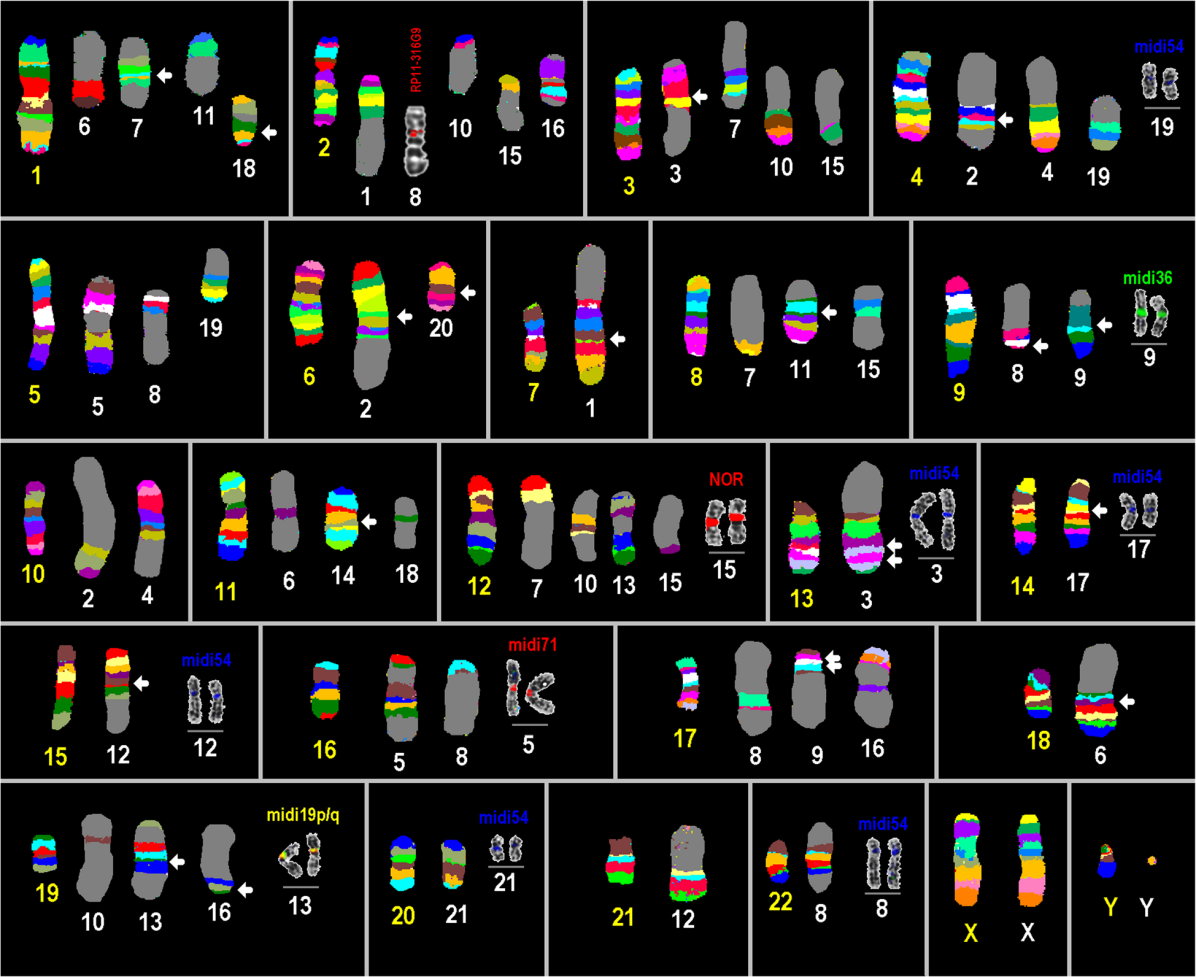
Representative MCB results: HPI in comparison to HSA chromosomes are shown as pseudo-color results. HSA chromosomes are numbered by yellow figures, HPI chromosomes by white figures. The chromosomes are sorted according to the HSA-chromosomes. Additional FISH-results are shown also for corresponding HPI chromosomes, where necessary. *Arrows* highlight interchromosomal rearrangements. Abbreviations: midi = microdissection derived probe (consecutively numbered acc. to production or localization); NOR = nucleolus organizer region

Results of the present study are summarized in Tables 1 and 2. Representative results of MCB, as well as regions being homologous to acrocentric short arms in HSA, the NOR-region, the region 9p12/9q13 and 16q11.2 are shown in Fig. 1. Also one locus-specific probe based FISH result for a region being homologous in HPI to human chromosome 2 can be found there. The latter is shown, as this region/rearrangement could not be visualized by MCB.

**Table 1:** Homologous regions, centromere position and heterochromatic inserts observed in HPI compared to HSA chromosomes

HPI chr.#	HPI-chromosomes reported as derivatives of human chromosomes	Centromeric position
1	2qter- > 2q22.3::7q21.11- > 7p22.3::7q21.11- > 7qter	as in HSA7
2	6qter- > 6q15::6p10- > 6q15::4q13.1- > 4q10::4q13.1- > 4q26::10p12.1- > 10pter	as in HSA6
3	het:3q22.1- > 3p12.3::3p14.3- > 3p12.3::13p13- > 13q21.32::13q33.2- > 13q21.32::13q33.2- > 13qter	as in HSA13
4	10qter- > 10p12.1::4q26- > 4qter	as in HSA10
5	16qter- > 16q22.2::5q31.1- > 5q14.1::16p12.2- > 16q22.2::5q31.1- > 5qter	as in HSA16
6	18qter- > 18q10::18p11.32- > 18p10::11p10- > 11q13.1::1q23.1- > 1p31.1:	as in HSA11 / 18
7	12pter- > 12p11.21::1q25.2- > 1q23.1::1p33- > 1p35.2::3p14.3- > 3p26.3::8p22- > 8pter	neo 12p11.21 / 1p35.3
8	16pter- > 16p12.2::5q14.1- > 5p10::2p11.2- > 2q10::22p13- > 22q13.33::17p13.3- > 17p11.2::9p24.3- > 9p12::9p24.3- > 9pter	as in HSA22
9	:17q22- > 17q23.2::17q21.1- > 17q10::17q21.1- > 17q22::9q21.12- > 9p12::9q21.12- > 9qter	as in HSA9
10	2pter- > 2p22.3::19q13.12- > 19q13.31::12q13.1- > 12p11.21::3q24- > 3qter	as HSA12
11	1pter- > 1p35.2::8q21.11- > 8p10::8q21.11- > 8qter	as in HSA8
12	:15q22.1- > 15p13::15q22.1- > 15q26.3::21q10- > 21qter	as in HSA15
13	19qter- > 19q13.42::12q22- > 12q13.3::19q13.12- > 19p13.2::19q13.32- > 19q13.42::12q22- > 12qter	as in HSA19
14	11qter- > 11q13.1::11p15.5- > 11p11.2:	neo 11q13.1 / 11p15.5
15	:2q22.3- > 2q14.2::NOR::8q10- > 8p22::3q22.1- > 3q24::12q13.3- > 12q13.1:	as in HSA8
16	17qter- > 17q23.2::2q14.2- > 2q10::17p11.2- > 17q11.2::2p11.2- > 2p22.3::19q13.31- > 19q13.32::19p13.2- > 19pter	as in HSA17
17	:14q21.2- > 14p13::14q21.2- > 14qter	as in HSA14
18	:1q32.2- > 1q25.2::11p10- > 11p11.2::1p33- > 1p31.1::1q32.2- > 1qter	as in HSA11
19	5pter- > 5q10::4p10- > 4pter - in centromere midi54+	as HSA4
20	:6p21.2- > 6q10::6p21.2- > 6pter	as in HSA6
21	20pter- > 20qter in centromere midi54+	as in HSA20
X	Xpter- > Xqter	as in HSAX
Y	Ypter- > Yqter	as in HSAY

*Abbreviations: #* number, *het* heterochromatin, *neo* neocentromere, *midi54+ *signal of probe homologous to acrocentric short arms in HSA

**Table 2:** The 68 ECBs detected in this study in HPI given as cytoband and genomic data (GRCh37/hg19) as well as FISH-probes used and comparison to HLA

Breakpoint (cytoband)	Narrowed down by	Localization acc. to GRCh37/hg19	Same ECB in HLA [8]
1p35.2	RP4-669K10 / RP11-114B7	28,853,741–33,101,404	+
1p33	RP11-330M19 / RP4-631H13	48,285,909–53,304,823	+
1p31.1	RP4-759M20 / RP5-944F13	67,033,180–70,103,142	+
1q23.1	RP11-307C12 / RP11-343F16	154,965,587–164,006,044	+
1q25.2	RP5-990P15 / RP11-181K3	178,490,946–183,063,114	+
1q32.2	RP11-110E24 / RP11-434B7	210,211,975–213,224,588	+
2p22.3	RP11-23B13 / RP11-119B15	30,979,722–35,864,069	+
2p11.2	RP11-316G9 / RP11-708D7	90,202,000–95,617,775	+
2p11.1 ~ q11.1	n.a.	90,500,000–96,800,000	-
2q14.2	Proximal to RP11-69O6	~110,000,000–121,987,648	+
2q22.3	RP11-107E5 / RP11-21M18	145,324,328–151,156,597	+
3p26.3	Proximal to subtel3pter	~2,000,000–~ 5,000,000	+
3p14.3	RP11-904G16 / RP11-229A12	54,646,599–57,395,394	-
3p12.3	RP11-552N9 / RP11-16M12	72,550,809–78,313,071	+
3q22.1	RP11-221E20 / RP11-517B11	128,695,100–131,245,291	
3q24	RP11-88H10 7 RP11-500K7	145,702,132–147,840,000	
4p11 ~ q11	n.a.	48,200,000–52,700,000	+
4q13.1	RP11-498E11 / RP11-92H22	66,083,151–71,660,470	-
4q26	MCB	126,000,000–137,000,000	+
5p11 ~ q11.1	n.a.	46,100,000–50,700,000	+
5q14.1	CTD-2200O3 / RP11-356D23	76,503,000–81,368,874	+
5q31.1	RP11-729C24 / CTD-2562E1	131,949,164–134,147,482	+
6p21.2	RP3-431A14	36,643,279–36,838,641	+
6p11.1 ~ q11	n.a.	58,700,000–63,300,000	+
6q15	RP11-223J24 / RP1-122O8	86,460,000–90,317,182	+
7p22.3	Distal to subtel7pter	0–~ 2,500,000	+
7q21.11	RP11-235F21 / RP11-448A3	76,680,916–81,315,990	+
8p22	RP11-19N21 / RP11-459H21	16,574,193–21,137,316	+
8p11.1 ~ q11.1	n.a.	43,100,000–48,100,000	+
8q21.11	RP11-347D13 / RP11-48D4	70,768,835–77,707,273	+
9p24.3	Proximal to subtel9pter	0–~ 2,000,000	+
9p12	Proximal to RP11-128P23	35,360,000–~ 50,000,000	+
9q21.12	Distal to RP11-373A9	72,849,245–~ 90,000,000	+
10p12.1	RP11-478H13 / RP11-379L21	23,279,768–29,095,050	+
11p15.5	Distal to subtel11pter	0–~ 1,200,000	+
11p11.2	Distal to RP11-397M16	48,150,000–~ 50,000,000	+
11p11.11-q11	n.a.	51,600,000–55,700,000	-
11q13.1	Distal to RP11-399J13	64,808,042–~ 70,000,000	+
12p11.21	RP11-517B23	31,471,644–32,029,051	-
12q13.1	Proximal to RP11-112N23	~45,000,000–50,731,377	+
12q13.3	RP11-112N23 / RP11-629N8	50,912,474–65,153,301	+
12q22	RP11-24I19 / RP11-406H4	94,634,754–99,487,137	+
13p13	n.a.	0–~ 200,000	-
13q21.32	RP11-100C24 / RP11-187E23	57,831,960–67,194,978	-
13q33.2	RP11-564N10 / RP11-141M24	102,655,660–109,369,625	+
14p13	n.a.	0–~ 200,000	-
14q21.2	RP11-35B20 / RP11-262M8	45,887,918–52,697,235	+
15p13	n.a.	0–~ 200,000	-
15q22.1	RP11-215J7 / RP11-219B17	55,027,961–60,973,768	+
15q26.3	Distal to subtel15qter	~102,200,000–102,531,392	+
16p12.2	RP11-705C1	24,089,175–24,270,169	-
16q22.2	RP5-991G20 / RP11-24I3	72,825,522–77,786,210	+
17p13.3	Proximal to subtel17pter	0–~ 500,000	+
17p11.2	Distal from RP11-746M1	~15,000,000–21,160,776-28	+
17q11.2	Distal from RP11-403E9	28,495,981–~ 40,000,000	-
17q21.1	RP11-47L3 / RP11-58O9	33,661,870–38,501,211	+
17q22	RP5-843B9 / RP11-429O1	46,228,000–50,467,875	+
17q23.2	RP11-142B17 / RP11-74H8	56,840,764–64,676,149	+
18p11.32	Proximal to subtel18pter	0–~ 200,000	+
18p11.1 ~ q11.1	n.a.	15,400,000–19,000,000	-
19p13.2	RP11-565J3 / RP11-79F15	6,979,038–8,853,332	+
19q13.12	RP11-430N3 / RP11-649P22	36,673,365–38,450,859	+
19q13.31	RP11-537N4 / RP11-21J15	40,997,000–45,034,762	+
19q13.32	RP11-21J15 / RP11-1089K2	45,208,382–47,311,583	+
19q13.42	RP11-10I11 / RP11-44L20	51,622,674–53,472,377	+
21p11.1 ~ q11.1	n.a.	10,900,000–14,300,000	+
22p13	n.a.	0–~ 200,000	-
22q13.33	Distal to subtel22qter	~51,000,000–51,304,566	+

As shown in Table 1, most HPI chromosomes are composed from regions being homologous to two or more HSA chromosomes; only HPI chromosomes X, Y, 14, 17 and 20 are homologous to only one human chromosome, each. Of those only chromosomes X, Y and 20 do not present gross chromosomal rearrangements according to FISH.

In Table 2 the 68 detected ECBs are compared with previous results from HLA. Interestingly only 11/68 ECBs in HPI were different from HLA.

Application of all human repetitive probes for heterochromatic regions (apart from centromeric ones) revealed that there were no homologies present for sequences derived from human Yq12, 1q12, 9q12, and 15p12 ~ 11.2. However, the same heterochromatic sequences being present at HSA 16q11.2 (midi71) were detected at HPI 5, those in HSA 9p12/9q13 (midi36) as one block on HPI 9, those in HSA 19p12 ~ 19q12 as one block on HPI 13, and those in human acrocentric short arms (midi54) on HPI 3, 8, 12, 17, 19, 21. NOR specific signals were only obtained as one block on both homologues of HPI 15. Finally, a block of HLA specific heterchromatin was present at end of HLA 3p.

The centromeric positions could be determined for all HPI chromosomes (Table 1). Interestingly, 20/23 positions are the same as in HSA. Centromeric positions of HSA chromosomes 11 and 18 are present at HPI 6 centromere. At HPI chromosomes 7 and 14 neocentromers are formed.

## Discussion

Here the first molecular cytogenetic study in HPI is presented using FISH-banding, locus-specific and human heterochromatic probes. It could be confirmed that MCB is well suited to gain a genome wide view on ECBs rapidly also in a yet little studied species (for review on comparable studies see [18]) (Fig. 1). Further narrowing down of ECBs can then be easily done using selected locus-specific probes (Tables 1 and 2).

In general, HPI has, compared to human, a highly rearranged karyotype. However, considering changes like translocations and inversions in HLA, chromosomes of HPI are therefore less affected (see Table 2 and [8]). On the other hand heterochromatic regions underwent in HPI a much faster evolution than in HLA; direct comparison is only possible for the regions being present in HSA as acrocentric short arms (midi54-probe) [19]: on the one hand midi54-positive signals are detectable in HLA only at one spot, in HPI at 6 different locations. On the other hand in HLA this DNA amplified at a region homologous to HSA 2, in HPI midi54-specific DNA seeded at regions homologous to centromeric positions of HSA chromosomes 4, 5, and all acrocentric short arms apart from HSA 21p. As homologous regions to HSA 15 and 21 are together on HPI chromosome 12, this also could be due to a fusion of both midi54-positive regions before an inversion in this chromosome took place.

At least 57/68 ECBs detected in HPI here are homologous to such detected in HLA. As 4/11 “new ECBs” compared to HLA are in terminal regions of HSA acrocentric short arms (Table 2), regions not present in HLA there, this may reduce the number of ECBs being really different in HPI and HLA to seven within the euchromatic sequences of HPI.

In this study the results obtained from application of heterochromatic probes suggest that chromosomal evolution in HPI was concentrated to these genomics regions, while in HLA euchromatin was hit more frequently. Both events might have been triggered by the recently suggested “propensity for a gibbon-specific retrotransposon (LAVA) to insert into chromosome segregation genes and alter transcription by providing a premature termination site, suggesting a possible molecular mechanism for the genome plasticity of the gibbon lineage” [8]. This, and the fact that centromeric positions changed in 13/23 chromosomes in HLA [19] and only in 3/23 in HPI needs to be elaborated by future studies.

## Conclusions

Overall, this study highlights that to study and understand genomes of other species, e.g. in comparison to human, it is necessary first to have the karyotypic data and then to combine this with more sophisticated ones, like next generation sequencing data. The latter approach is due to technical restrictions principally not able to detect the peculiarities of heterochromatic regions, while molecular cytogenetic approaches cannot provide a basepair-resolution. Thus, only the combination of all available technical means will help us to understand the miracles of evolution and nature in the end.

## Methods

### Cell culture and chromosomal preparation

Immortalized lymphoblast cell lines derived from a male HPI, was provided by the Khon Kaen University, Thailand. Culture techniques as well as chromosome preparation followed standard protocols.

### Fluorescence in situ hybridization

All protocols for FISH have been provided in our previous study by us in Fan et al. [18]; details on MCB, single and dual-color FISH techniques which were performed for the applied bacterial artificial chromosome (BAC-) probes and the commercially derived subtelomeric probes can be found there. All here used BAC-probes are listed in Additional file 1: Table S1. The homemade heterochromatin mix (HCM-) probe set [20] was also described there [18] covering chromosomal regions 1q12, 16q11.2 (midi71), 9q12, 9p12/ 9q13 (midi36) 15p11.2-p11.1, all acrocentric short arms (midi54), 19p12/q12 and Yq12.

## Additional file

Additional file 1:
**The BAC probes applied in the present study.** Abbreviations: n.a. = not available; subtel = subtelomeric probe. (DOC 165 kb)

## Abbreviations

ECBs: evolutionary conserved breakpoints
FISH: fluorescence in situ hybridization
*H.*: *Hylobates*
HAG: *Hylobates agilis*
HLA: *Hylobates lar*
HPI: *Hylobates pileatus*
HSA: *Homo sapiens*
MCB: multicolor banding
midi: microdissection derived probe
NOR: nucleolus organizer region
