# Supplementary material for: Application of multicolor banding combined with heterochromatic and locus-specific probes identify evolutionary conserved breakpoints in Hylobates pileatus
Source: Mol Cytogenet. 2016 Feb 17;9:17. doi: 10.1186/s13039-016-0228-x (PMC4758170; doi:10.1186/s13039-016-0228-x)
Supplement: Additional file 1: — The BAC probes applied in the present study. Abbreviations: n.a. = not available; subtel = subtelomeric probe. (DOC 165 kb) [file 13039_2016_228_MOESM1_ESM.doc]

| **BAC name** | **Cytoband** | **Localization acc. to GRCh37/hg19** |
| --- | --- | --- |
| RP3-398I9 | 1p36.11 | chr1:25,191,442-25,286,682 |
| RP11-335G20 | 1p36.11 | chr1:25,688,740-25,747,363 |
| RP4-669K10 | 1p35.3 | chr1:28,852,835-28,853,741 |
| RP11-114B7 | 1p35.1 | chr1:33,101,404-33,274,280 |
| RP11-342M1 | 1p34.2 | chr1:43,314,962-43,318,146 |
| RP11-330M19 | 1p33 | chr1:48,283,738-48,285,909 |
| RP4-631H13 | 1p32.3 | chr1:53,304,823-53,421,058 |
| RP4-759M20 | 1p31.3 | chr1:66,904,686-67,033,180 |
| RP5-944F13 | 1p31.1 | chr1:70,103,142-70,216,656 |
| RP11-297N6 | 1p31.1 | chr1:76,046,221-76,227,510 |
| RP4-653E17 | 1p31.1 | chr1:82,070,192-82,125,867 |
| RP11-196D4 | 1q21.3 | chr1:154,756,481-154,933,673 |
| RP11-307C12 | 1q21.3 | chr1:154,960,533-154,965,587 |
| RP11-343F16 | 1q23.3 | chr1:164,006,044-164,196,440 |
| RP11-160H22 | 1q25.1 | chr1:173,949,371-174,121,279 |
| RP5-990P15 | 1q25.2 | chr1:178,482,212-178,490,946 |
| RP11-181K3 | 1q25.3 | chr1:183,063,114-183,227,197 |
| RP11-152M20 | 1q32.1 | chr1:199,508,362-199,647,085 |
| RP11-739N20 | 1q32.1 | chr1:204,346,978-204,404,974 |
| RP11-430C7 | 1q32.1 | chr1:204,572,163-204,585,693 |
| RP11-328D5 | 1q32.2 | chr1:208,059,883-208,063,837 |
| RP11-1126G17 | 1q32.2 | chr1:209,651,580-209,794,478 |
| RP11-110E24 | 1q32.2 | chr1:210,053,186-210,211,975 |
| RP11-434B7 | 1q32.3 | chr1:213,224,588-213,446,808 |
| RP11-264D3 | 1q41 | chr1:217,634,630-217,788,033 |
| RP11-109G24 | 1q42.2 | chr1:234,264,342-234,420,603 |
| RP5-940F7 | 1q42.3 | chr1:236,089,034-236,192,641 |
| RP11-553N16 | 1q43 | chr1:241,935,720-242,079,686 |
|  |  |  |
| RP11-23B13 | 2p23.1 | chr2:30,812,078-30,979,722 |
| RP11-119B15 | 2p22.3 | chr2:35,864,069-36,032,088 |
| RP11-288C18 | 2p22.2 | chr2:37,051,500-37,132,046 |
| RP11-299C5 | 2p21 | chr2:42,480,290-42,665,241 |
| RP11-316G9 | 2p11.2 | chr2:89,973,000-90,202,000 |
| RP11-708D7 | 2q11.2 | chr2:95,617,775-95,787,635 |
| RP11-69O6 | 2q14.2 | chr2:121,987,648-122,159,459 |
| RP11-707C13 | 2q14.3 | chr2:125,559,040-125,711,820 |
| RP11-89B17 | 2q21.1 | chr2:132,280,845-132,455,004 |
| RP11-289K3 | 2q21.3 | chr2:135,922,853-136,074,755 |
| RP11-15D9 | 2q22.1 | chr2:139,736,474-139,903,043 |
| RP11-107E5 | 2q22.3 | chr2:145,164,538-145,324,328 |
| RP11-21M18 | 2q23.3 | chr2:151,156,597-151,331,640 |
|  |  |  |
| RP11-189H19 | 3p21.32-3p21.31 | chr3:44,460,437-44,890,926 |
| RP11-78O10 | 3p21.31 | chr3:49,223,538-50,031,884 |
| RP11-904G16 | 3p14.3 | chr3:54,448,784-54,646,599 |
| RP11-229A12 | 3p14.3 | chr3:57,395,394-57,559,893 |
| RP11-522N9 | 3p13 | chr3:72,372,591-72,550,809 |
| RP11-16M12 | 3p12.3 | chr3:78,313,071-78,482,308 |
| RP11-333I20 | 3p12.3 | chr3:78,680,000-79,090,00 |
| RP11-382L10 | 3p12.1 | chr3:83,530,000-83,920,000 |
| RP11-59J16 | 3q21.3 | chr3:127,116,770-127,269,356 |
| RP11-221E20 | 3q21.3 | chr3:128,509,053-128,695,100 |
| RP11-517B11 | 3q22.1 | chr3:131,245,291-131,246,023 |
| RP11-48F15 | 3q22.1 | chr3:132,339,897-132,476,540 |
| RP11-269A14 | 3q22.3 | chr3:136,749,172-136,934,234 |
| RP11-88H10 | 3q24 | chr3:145,528,679-145,702,132 |
| RP11-500K7 | 3q24 | chr3:147,840,000-148,400,000 |
| RP11-251C9 | 3q25.1 | chr3:150,617,581-150,797,073 |
| RP11-111F10 | 3q25.31 | chr3:155,630,550-155,803,492 |
|  |  |  |
| RP11-498E11 | 4q13.1 | chr4:66,082,334-66,083,151 |
| RP11-92H22 | 4q13.3 | chr4:71,660,470-71,814,886 |
|  |  |  |
| RP11-103A15 | 5q12.1 | chr5:60,511,679-60,666,928 |
| RP11-480H11 | 5q12.3 | chr5:65,023,404-65,203,264 |
| RP11-164H10 | 5q13.2 | chr5:70,144,725-70,311,057 |
| CTD-2200O3 | 5q14.1 | chr5:76,169,000-76,503,000 |
| RP11-356D23 | 5q14.1 | chr5:81,368,874-81,369,521 |
| RP11-321G16 | 5q23 | chr5:118,780,000-119,210,000 |
| CTC-461G12 | 5q23 | chr5:119,521,000-119,845,000 |
| RP11-48C14 | 5q23.2 | chr5:124,151,396-124,323,070 |
| RP11-394D6 | 5q23.2 | chr5:125,650,000-126,140,000 |
| RP11-434D11 | 5q23.2 | chr5:126,087,655-126,112,178 |
| RP1-179P12 | 5q23.3 | chr5:128,960,000-132,110,000 |
| RP11-729C24 | 5q31.1 | chr5:131,789,105-131,949,164 |
| CTD-2562E1 | 5q31.1 | chr5:134,147,482-134,360,279 |
|  |  |  |
| RP1-34B20 | 6p22.2 | chr6:26,194,847-26,284,147 |
| RP11-111A4 | 6p22.1 | chr6:26,841,085-26,999,223 |
| RP1-34F7 | 6p21.33 | chr6:31,923,944-32,092,846 |
| RP1-90K10 | 6p21.2 | chr6:36,907,847-36,912,451 |
| RP3-431A14 | 6p21.2 | chr6:36,643,279-36,838,641 |
| RP11-223L24 | 6q14.3 | chr6:84,216,000-84,369,000 |
| RP11-30P6 | 6q14.3 | chr6:85,995,794-86,000,567 |
| RP11-223J2 | 6q14.3 | chr6:86,030,000-86,460,000 |
| RP1-122O8 | 6q15 | chr6:90,317,182-90,457,390 |
| RP11-482L14 | 6q16.1 | chr6:95,830,553-96,039,148 |
| RP11-572N15 | 6q16.1 | chr6:95,696,000-96,009,000 |
|  |  |  |
| RP11-235F21 | 7q11.23 | chr7:76,558,528-76,680,916 |
| RP11-448A3 | 7q21.11 | chr7:81,315,990-81,511,944 |
| RP11-212B1 | 7q21.12 | chr7:87,068,238-87,216,464 |
|  |  |  |
| RP11-177H2 | 8p23.1 | chr8:10,596,865-10,759,085 |
| RP11-589N15 | 8p23.1 | chr8:11,589,980-11,765,702 |
| RP11-44L18 | 8p22 | chr8:15,513,190-15,655,319 |
| RP11-19N21 | 8p22 | chr8:16,399,691-16,574,193 |
| RP11-459H21 | 8p21.3 | chr8:21,137,316-21,344,395 |
| RP11-347D13 | 8q13.3 | chr8:70,593,909-70,768,835 |
| RP11-48D4 | 8q21.11 | chr8:77,707,273-77,870,908 |
| RP11-62E9 | 8q21.13 | chr8:81,741,781-81,933,128 |
| RP11-354A14 | 8q21.13 | chr8:82,789,445-82,975,674 |
| RP11-317J10 | 8q21.2 | chr8:86,263,108-86,444,141 |
|  |  |  |
| RP11-128P23 | 9p11.2 | chr9:42,460,000-45,360,000 |
| RP11-262H14 | 9q13 | chr9:66,553,276-66,555,594 |
| RP11-381O7 | 9q13 | chr9:66,913,511-67,107,560 |
| RP11-12P21 | 9q13 | chr9:68,209,003-68,372,456 |
| RP11-391M20 | 9q21.11 | chr9:68,726,629-68,745,997 |
| RP11-373A9 | 9q21.12 | chr9:72,804,135-72,849,245 |
| RP11-535C21 | 9q22.33 | chr9:100,748,833-100,749,938 |
| RP11-318L4 | 9q31.1 | chr9:106,317,536-106,339,433 |
| RP11-388N6 | 9q31.3 | chr9:112,178,807-112,187,409 |
|  |  |  |
| RP1-251M9 | 10p14 | chr10:11,002,114-11,012,779 |
| RP11-16O1 | 10p12.33 | chr10:17,775,718-17,881,746 |
| RP11-379L21 | 10p12.2 | chr10:23,083,774-23,279,768 |
| RP11-478H13 | 10p12.1 | chr10:29,095,050-29,102,387 |
|  |  |  |
| RP11-722K13 | 11p11.2 | chr11:47,523,040-48,006,704 |
| RP11-793I11 | 11p11.2 | chr11:47,926,875-47,927,159 |
| RP11-397M16 | 11p11.2 | chr11:48,150,000-48,590,000 |
| RP11-399J13 | 11q13.1 | chr11:64,781,654-64,808,042 |
|  |  |  |
| RP11-517B23 | 12p11.21 | chr12:31,471,644-32,029,051 |
| RP11-112N23 | 12q13.1 | chr12:50,731,377-50,912,474 |
| RP11-629N8 | 12q14.3 | chr12:65,153,301-65,173,098 |
| RP11-202H2 | 12q21.32 | chr12:86,993,355-87,232,775 |
| RP11-900F13 | 12q21.32-12q21.33 | chr12:88,850,430-89,022,675 |
| RP11-24I19 | 12q22 | chr12:94,470,882-94,634,754 |
| RP11-406H4 | 12q23.1 | chr12:99,487,137-99,498,789 |
| RP11-426H24 | 12q23.1-12q23.2 | chr12:101,418,480-101,616,644 |
|  |  |  |
| RP11-115I22 | 13q14.2 | chr13:49,003,160-49,167,781 |
| RP11-100C24 | 13q21.1 | chr13:57,702,596-57,831,960 |
| RP11-187E23 | 13q21.32 | chr13:67,194,978-67,366,336 |
| RP11-552M6 | 13q22.1 | chr13:74,282,718-74,404,613 |
| RP11-564N10 | 13q33.1 | chr13:102,654,475-102,655,660 |
| RP11-141M24 | 13q33.3 | chr13:109,369,625-109,543,362 |
| RP11-245B11 | 13q34 | chr13:114,873,044-114,874,295 |
|  |  |  |
| RP11-138H18 | 14q21.1 | chr14:38,267,455-38,418,018 |
| RP11-35B20 | 14q21.2 | chr14:45,706,083-45,887,918 |
| RP11-262M8 | 14q22.1 | chr14:52,697,235-52,895,024 |
|  |  |  |
| RP11-353B9 | 15q21.2 | chr15:49,896,865-50,081,728 |
| RP11-215J7 | 15q21.3 | chr15:54,865,291-55,027,961 |
| RP11-219B17 | 15q22.2 | chr15:60,973,768-60,979,087 |
|  |  |  |
| RP11-958N24 | 16p13.11-12.3 | chr16:16,251,898-16,483,256 |
| RP11-14N9 | 16p13.11-12.3 | chr16:16,723,020-16,856,594 |
| RP11-338J22 | 16p12.2 | chr16:21,613,952-21,791,482 |
| RP11-705C1 | 16p12.2-16p12.1 | chr16:24,089,175-24,270,169 |
| RP5-991G20 | 16q22.2 | chr16:72,823,142-72,825,522 |
| RP11-24I3 | 16q23.1 | chr16:77,786,210-77,981,585 |
| RP11-303E16 | 16q23.2 | chr16:81,105,603-81,110,203 |
| RP11-21B21 | 16q24.2 | chr16:88,579,368-88,597,461 |
|  |  |  |
| RP11-746M1 | 17p11.2 | chr17:20,878,522-21,160,776 |
| RP11-403E9 | 17q11.2 | chr17:28,495,981-28,669,176 |
| RP11-47L3 | 17q12 | chr17:33,499,406-33,661,870 |
| RP11-58O9 | 17q21.2 | chr17:38,501,211-38,667,419 |
| RP11-506G7 | 17q21.2~21.31 | chr17:40,886,393-41,074,128 |
| RP5-843B9 | 17q21.32 | chr17:45,912,000-46,228,000 |
| RP11-429O1 | 17q22 | chr17:50,467,875-50,613,061 |
| RP11-21M22 | 17q22 | chr17:56,594,306-56,729,669 |
| RP11-515O17 | 17q22 | chr17:53,299,235-53,481,282 |
| RP11-142B17 | 17q22 | chr17:56,666,963-56,840,764 |
| RP11-74H8 | 17q24.2 | chr17:64,676,149-64,816,307 |
|  |  |  |
| CTD-2589F14 | 19p13.3 | chr19:478,636-702,131 |
| RP11-500M22 | 19p13.3 | chr19:4,700,000-5,210,000 |
| RP11-565J3 | 19p13.3-19p13.2 | chr19:4,787,411-6,979,038 |
| RP11-79F15 | 19p13.2 | chr19:8,853,332-9,003,039 |
| CTD-3222D19 | 19p13.11 | chr19:16,676,362-16,677,141 |
| RP11-615P5 | 19q13.11 | chr19:34,681,165-34,866,767 |
| RP11-8N10 | 19q13.11 | chr19:35,302,005-35,472,810 |
| RP11-430N3 | 19q13.12 | chr19:36,472,309-36,673,365 |
| RP11-649P22 | 19q13.13 | chr19:38,450,859-38,627,682 |
| RP11-537N4 | 19q13.2 | chr19:40,443,000-40,997,000 |
| RP11-21J15 | 19q13.31 | chr19:45,034,762-45,208,382 |
| RP11-1089K2 | 19q13.32 | chr19:47,311,583-47,509,119 |
| RP11-521I20 | 19q13.33 | chr19:49,187,000-49,667,000 |
| RP11-264M8 | 19q13.33 | chr19:50,075,803-50,233,543 |
| RP11-369N17 | 19q13.33 | chr19:50,537,011-50,652,984 |
| RP11-10I11 | 19q13.41 | chr19:51,469,617-51,622,674 |
| RP11-44L20 | 19q13.41-13.42 | chr19:53,472,377-53,644,819 |
| RP11-155P5 | 19q13.42 | chr19:55,217,411-55,391,988 |
| CTC-550B14 | 19q13.42 | chr19:55,538,961-55,565,296 |
